# Quantitative decision making for investment in global health intervention trials: Case study of the NEWBORN study on emollient therapy in preterm infants in Kenya

**DOI:** 10.7189/jogh.12.04045

**Published:** 2022-06-11

**Authors:** Annie Stylianou, Keona J H Blanks, Rachel A Gibson, Lindsay K Kendall, Mike English, Sarah Williams, Roshni Mehta, Andrew Clarke, Lynn Kanyuuru, Jalemba Aluvaala, Gary L Darmstadt

**Affiliations:** 1 GlaxoSmithKline R&D, Gunnels Wood Road, Stevenage, Hertfordshire, UK; 2 Stanford University, Stanford, California, USA; 3 Oxford Centre for Global Health Research, Nuffield Department of Clinical Medicine, Oxford, UK; 4 KEMRI-Wellcome Trust Research Programme, Nairobi, Kenya; 5 Save the Children UK, London, UK; 6 Save the Children International, Kenya Country Office, Nairobi, Kenya; 7 Department of Paediatrics and Child Health, University of Nairobi, Nairobi, Kenya; 8 Department of Pediatrics, Stanford University School of Medicine, Stanford, California, USA

## Abstract

**Background:**

Partners from an NGO, academia, industry and government applied a tool originating in the private sector – Quantitative Decision Making (QDM) – to rigorously assess whether to invest in testing a global health intervention. The proposed NEWBORN study was designed to assess whether topical emollient therapy with sunflower seed oil in infants with very low birthweight <1500 g in Kenya would result in a significant reduction in neonatal mortality compared to standard of care.

**Methods:**

The QDM process consisted of prior elicitation, modelling of prior distributions, and simulations to assess Probability of Success (PoS) via assurance calculations. Expert opinion was elicited on the probability that emollient therapy with sunflower seed oil will have any measurable benefit on neonatal mortality based on available evidence. The distribution of effect sizes was modelled and trial data simulated using Statistical Analysis System to obtain the overall assurance which represents the PoS for the planned study. A decision-making framework was then applied to characterise the ability of the study to meet pre-selected decision-making endpoints.

**Results:**

There was a 47% chance of a positive outcome (defined as a significant relative reduction in mortality of ≥15%), a 45% chance of a negative outcome (defined as a significant relative reduction in mortality <10%), and an 8% chance of ending in the consider zone (ie, a mortality reduction of 10 to <15%) for infants <1500 g.

**Conclusions:**

QDM is a novel tool from industry which has utility for prioritisation of investments in global health, complementing existing tools [eg, Child Health and Nutrition Research Initiative]. Results from application of QDM to the NEWBORN study suggests that it has a high probability of producing clear results. Findings encourage future formation of public-private partnerships for health.

International public-private partnerships for health (PPPH) have become increasingly important for advancing public health in low- and middle-income countries (LMICs) [1]. The pharmaceutical industry contributes substantially to global health programs each year, with inputs extending well beyond monetary resources [2]. According to the World Economic Forum, PPPHs have the potential to maximise health benefits for the poor and minimise potential risks for the partners involved, with true partnerships combining different skills, expertise, and resources to achieve a common goal more effectively than by independent action [3]. The current paper illustrates an approach implemented through a PPPH for critically evaluating investments to expand the evidence base for interventions and accelerate impact in global health.

The non-governmental organisation (NGO), Save the Children; the private sector pharmaceutical company, GlaxoSmithKline (GSK); the academic institutions, Stanford University and Oxford University; and the Kenya Medical Research Institute (KEMRI)-Wellcome Trust, a country-based governmental body responsible for carrying out health research in Kenya, came together to propose a study in Kenya to investigate whether benefits of topical emollient therapy seen in other settings, particularly in South Asia, can be extended to the East African context. Leveraging expertise present in the public and private sectors, we sought to investigate the potential for emollient therapy to address leading causes of neonatal mortality – prematurity and serious infections – in low- and middle-income countries (LMICs) [4].

Very preterm (<32 week gestational age) and very low birth weight (VLBW) infants are particularly vulnerable to mortality, in part due to under-developed skin barrier function [5]. The skin barrier of very preterm infants is lacking in vernix, a naturally protective cutaneous biofilm [6], and is easily injured and functionally compromised [7]. The stratum corneum does not fully develop until late in the third trimester, leaving very preterm neonates inadequately protected from fluid and heat loss and the entry of microbial pathogens. This problem is compounded in LMICs by heavy loads of virulent pathogens in unsterile environments [8] as well as maternal and newborn malnutrition [9]. KEMRI found that the majority (>95%) of newborn infants die of preventable causes, with most originating from a lack of access to basic high-quality health services including essential facility-based inpatient care [10]. Risk for mortality is particularly high – approximately 50% – among VLBW infants in Kenya [10-13].

Several studies have reported that the frequent application of certain topical emollients (eg, sunflower seed oil) to the skin of preterm infants may significantly improve growth and has the potential to reduce hospital-acquired infection and mortality [14]. However, such data are scarce from sub-Saharan Africa [15]. Through a research and implementation collaboration developed at the intersection of industry, NGOs, academia, and government, our PPPH aimed to ensure an end-to-end approach to investigation and integration of evidence-based emollient therapy in the routine care of preterm infants in LMICs in sub-Saharan Africa.

The present paper applied the Quantitative Decision Making (QDM) framework created by GSK to quantitatively assess the probability of success (PoS) for an intervention trial to answer a given research question [16]. This methodology was adopted to assess the probability that the Neonatal Emollient for faster Weight-gain, Better Outcomes, Reduced mortality and Nosocomial infection (NEWBORN) Study will demonstrate a significant reduction in neonatal mortality and will represent a sound investment in public health impact.

## METHODS

### The QDM process

#### Introduction

The QDM framework was created by GSK to quantitatively assess the PoS of planned investments in research trials on products under development. The QDM process consists of prior elicitation, modelling of prior distributions (the design prior or the uncertainty around the true unknown treatment effect), and simulations of clinical trials to assess PoS via assurance calculations [17]. The use of assurance calculations to inform the design of studies, communicate PoS, and aid investment decision-making is now applied regularly to research projects at GSK.

#### Elicitation of prior distributions: synthesising current knowledge

Incorporating a prior distribution into decision-making processes enables the utilisation of all available knowledge around the true treatment effect in PoS calculations. This is based on clinical experience and data from previous similar studies. Prior distribution is informed by a formal prior elicitation process where a number of key experts in the research field of interest are consulted to elicit the best available judgements from a clinical and scientific perspective [18]. Conducting a prior elicitation is a formal process, ideally conducted face-to-face if possible, whereby experts are asked a set of questions around their beliefs regarding one or more uncertain quantities (eg, treatment effect). Following a formal prior elicitation process, the statistician will derive a probability distribution for the quantity of interest which needs to reflect the expert beliefs about the value of the quantity as well as the uncertainty around that belief (the design prior) [19].

#### Simulation of assurance and estimation of probability of study success

The concept of assurance was first advanced by O’Hagan et al. [17] to incorporate all available knowledge around the true treatment effect in order to quantify the PoS of a proposed study. This approach relies on quantifying available knowledge using a probability distribution which represents the uncertainty around the unknown true treatment effect.

A key consideration for a study sponsor when designing a trial is the power of the study, which is driven by numerous factors including the sample size, the hypothesis to be tested and the significance level (α). The sample size is chosen to ensure the study will provide sufficient power (the probability of achieving statistical significance assuming a true effect is present) and is usually chosen to be 80 or 90%. A key limitation with power calculations is the need to assume a fixed true value for the treatment effect. Power does not represent the probability of a study having a successful outcome, since at the planning stage the true underlying treatment effect is unknown. Power is merely the conditional probability of a successful trial – it is conditional on the unknown assumed treatment effect used in the power calculations at the planning stage.

Assurance, on the other hand, considers a collection of available evidence around the treatment effect when designing a trial [20]. Unlike power, assurance represents the unconditional probability that a trial will result in a specific outcome, taking into account the uncertainty around the true unknown treatment effect (known as the prior) and is therefore of greater potential value in the planning of trials. In the approach taken by O’Hagan, the uncertainty from the sampling variability is part of the power function, which is predictive conditional on a fixed value of the true treatment difference, Δ. The assurance is calculated as [17,21]:



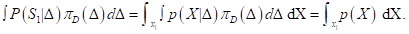



Where

Δ represents the true treatment difference,

*π_D_* (Δ) represents the design prior for the true treatment difference,

*X* denotes the data which has the likelihood *p* (*X* |Δ),

*S*_1_ represents the event of achieving a pre-defined success criterion.

Simulation techniques can then be utilised to calculate the assurance, in order to obtain an estimate of the PoS by incorporating the sampling from the prior distribution. This process, known as Bayesian clinical trial simulation, involves three key steps. First, simulation is used to sample a value from the prior distribution. Then simulation is used to sample the outcome of the trial based on the prior distribution and the characteristics of the planned study design such as proposed sample size and size of the pre-defined clinically relevant difference. Finally, an assessment is made whether the simulated trial will produce positive or negative results. These steps are repeated numerous times (eg, 1 000 000 simulated trials) and then the PoS is derived based on the number of simulated trials which are positive divided by total number of simulated trials.

### Proposed NEWBORN Study Design

The proposed NEWBORN study was designed to assess whether topical emollient therapy with high-linoleate (>60% linoleic acid) sunflower seed oil in preterm infants would result in a significant reduction in neonatal mortality compared to standard of care. A relative reduction of ≥15% was considered significant and could warrant policy change for adoption of emollient therapy for VLBW infants in Kenyan hospitals. The study planned to recruit babies weighing ≤1500 g (or ≤2000 g) – with VLBW (<1500 g) as a proxy for being very preterm, approximating a gestational age of <32-33 weeks) – and to randomise eligible babies to the emollient therapy or control (standard of care) arms in a 1:1 ratio. The primary endpoint was all-cause mortality (absolute count of deaths) based on a difference of morality rates of the two study groups assessed up to the time of discharge from the hospital or death within 28 completed days (ie, the end of the neonatal period), whichever occurred sooner. The study was due to be carried out in Kenya where the neonatal mortality rate in infants ≤1500 g is estimated to be about 50% (and 30% for newborn infants ≤2000 g) [10-13,22]. While many interventions are recommended by the WHO for use in VLBW in settings such as Kenya [23,24], quality of care and availability of many life-saving interventions is variable [25-27]. Emollient therapy is an inexpensive intervention that can be readily applied and appears to be acceptable for use by frontline workers and families, and thus has potential for widespread use [28,29].

### Application of QDM to the NEWBORN study

#### Elicitation of prior distribution

In applying the QDM process to the NEWBORN study, we aimed to address two key questions as part of the prior elicitation. First, what is the probability that any planned emollient intervention would have any benefit above standard of care? Second, assuming the planned intervention does have some benefit (above a control arm/standard of care), then what is the range of plausible effects?

In order to address these requirements of the QDM process, a panel of experts was formed which consisted of GSK personnel, external experts and key opinion leaders in maternal and newborn health; three panel members have particular expertise in newborn care in low-resource settings (Table S1 in the **Online Supplementary Document**). The second step was to prepare a package of available evidence within this field which was comprised of data from previous study publications assessing emollient therapy and neonatal mortality (Table S2 in the **Online Supplementary Document**). This package of information was shared with the panel and the following two specific questions were posed for the panel’s consideration based on their expert knowledge in this field and the available package of data: 1) “What do you feel is the probability that emollient therapy will have any measurable benefit on neonatal mortality above standard of care?” and 2) “Assuming emollient therapy has some measurable benefit on mortality, what do you believe are the plausible range of values (e.g., lower bound and upper bound) for relative reduction in neonatal mortality? The plausible range should represent the range of values that you are 99% certain the “true” relative reduction lies within – in other words, you would be very surprised if the “true” reduction was outside of this range.” The panel was asked to consider these two questions based on their knowledge of newborn infants who are A)≤1500 g, and B)≤2000 g. As it was not possible to hold a face-to-face prior elicitation, experts were asked to provide a written justification for their beliefs.

#### Estimation of prior distribution

A bi-modal distribution was adopted for the design prior in order to (i) account for the probability that emollient therapy has no measurable benefit in neonatal mortality above standard of care, and (ii) account for the probability that emollient therapy has some measurable benefit above standard of care with some uncertainty around the true effect size. This was achieved by eliciting the experts’ opinions on the probability that the treatment has a true positive/favourable effect (‘w’), and eliciting the minimum and maximum range of the relative reduction in neonatal mortality under the assumption that the treatment does have a favourable effect. A bi-modal prior was then formed to represent the overall prior for the treatment effect by (i) utilising a uniform distribution to model the absolute reduction in mortality (derived from the elicited minimum and maximum effect range for the relative reduction), and weighting this distribution by ‘w’, and (ii) utilising a normal distribution to introduce a ‘spike’, with weight ‘1-w’, at zero to model the distribution of a treatment effect, in line with standard of care, (ie, represents no effect of emollient therapy) [19].

#### Simulation of assurance and estimation of PoS

Statistical Analysis System was utilised to simulate the results of 1 000 000 trials, designed to detect a statistically significant relevant reduction of ≥15%, using the bi-model design prior. In order to weight the uniform and normal distributions accordingly within the bi-model, data was initially simulated from a standard discrete uniform distribution U (0,1). If the simulated value was ≤’w’, then trial results were simulated from a uniform distribution U (a, b) which represents a favourable effect accounting for the minimum absolute reduction (a) and maximum absolute reduction (b) in mortality. If the simulated value was>’w’, then trial results were simulated from a normal distribution, N (μ,σ^2^) where μ is the mean reduction in mortality due to emollient therapy in line with standard of care (ie, represents no effect) and σ^2^ represents a small variance around the mean. Similar simulations were run to assess the bi-modal design prior distribution for infants ≤2000 g.

Success, based on observing a statistically significant relative reduction of ≥15% in neonatal mortality, was then derived for each of these simulated trials. The overall assurance was estimated based on the number of simulated trials with a statistically significant reduction of ≥15% in mortality over the total number of simulated trials. This overall assurance is then a representation of the PoS for the planned trial.

#### Decision-making framework

Decision-making endpoints for this study were based on reductions in mortality. Key secondary endpoints were weight gain, time to hospital discharge and infection rates. Other non-decision-making endpoints included clinical improvement of skin condition based on clinical assessment scores and reductions in rates of transepidermal water loss (TEWL) as a measure of skin barrier integrity.

A positive outcome was defined as a statistically significant relative reduction in all-cause neonatal mortality and on observing a ≥15% relative reduction following emollient therapy compared to standard of care. A negative outcome was defined as a <10% relative reduction in all-cause neonatal mortality following emollient therapy compared to standard of care. We further defined a “consider zone” as a mortality reduction of 10 to <15%, which would result in consideration of key secondary endpoints including weight gain, time to discharge, physician reported infection rates, skin condition and TEWL.

### Patient and public involvement

Patients/the public were not involved in this study.

## RESULTS

### Prior elicitation

**Figure 1:**
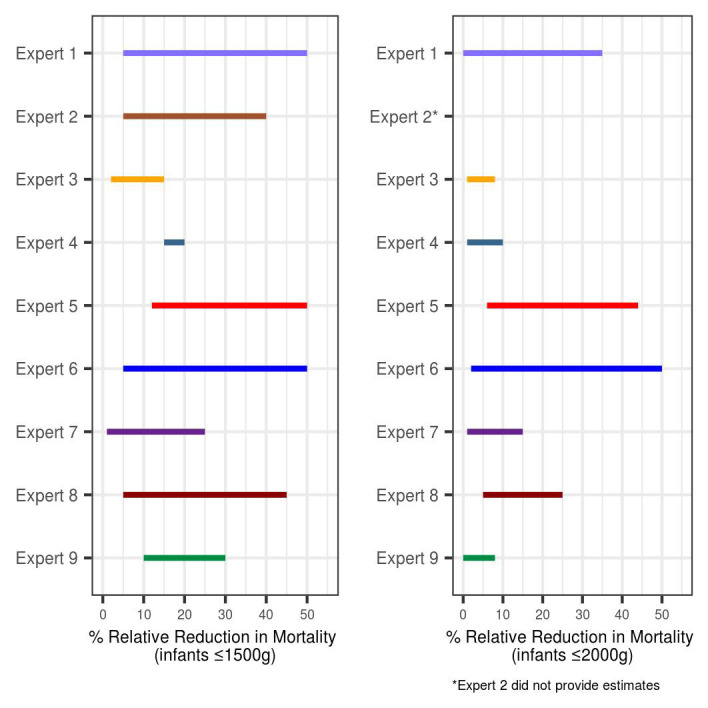
Bi-modal design prior distribution based on reduction in neonatal mortality in infants. **Panel A.** Infants ≤1500 g. **Panel B.** Infants ≤2000 g.

The overall consensus from the QDM panel following the prior elicitation process was that there was a 66% probability that emollient intervention would have some benefit on mortality (and 34% probability that there would be no benefit), above standard of care in infants ≤1500 g (**Figure 1**, Panel A). For infants born weighing ≤2000 g, the panel placed 48% weight, on average, on the probability of emollient therapy having some benefit and 52% probability of no benefit, above standard of care (**Figure 1**, Panel B).

**Figure 2:**
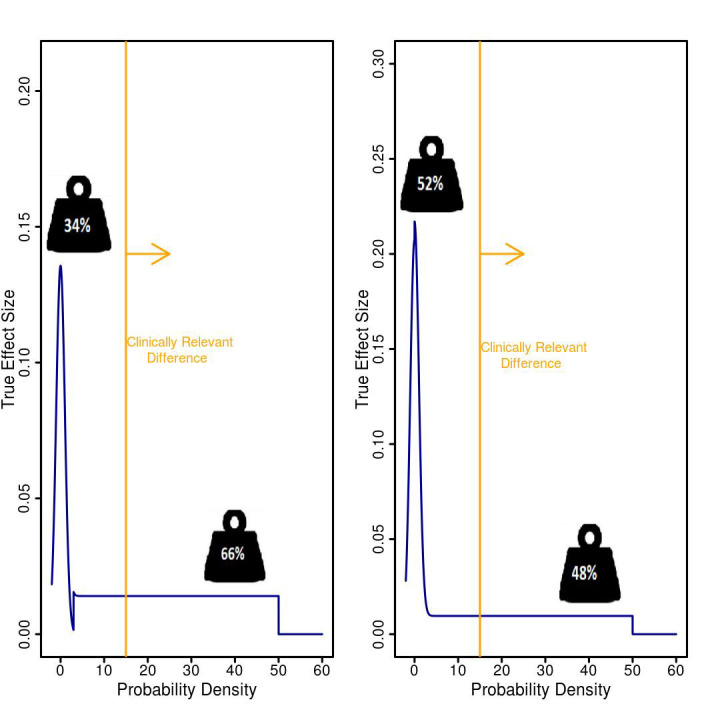
Expert panel member estimates of the plausible range of mortality reduction from emollient therapy in infants. **Panel A.** Very low birth weight infants ≤1500 g* **Panel B.** Infants ≤2000 g* Expert panel members were asked: “Assuming emollient therapy has some measurable benefit on mortality, what do you believe are the plausible range of values (e.g., lower bound and upper bound) for relative reduction in neonatal mortality?”

Assuming emollient therapy has some benefit on neonatal mortality, the panel elicited the effect of emollient therapy to be in the range of 1%-50% above standard of care for infants ≤1500 g (**Figure 2**, Panel A) and for infants ≤2000 g (**Figure 2**, Panel B).

### Simulations

SAS simulation code for the bi-modal design prior distribution is shown in Table S3A in the **Online Supplementary Document** for infants ≤1500 g, and in Table S4A in the **Online Supplementary Document** for infants ≤2000 g. Trial simulations and output from trial simulations for infants ≤1500 g are shown in Tables S3B and S3C in the **Online Supplementary Document**, respectively. Corresponding trial simulations and output for infants ≤2000 g are shown in Tables S4B and S4C in the **Online Supplementary Document**, respectively. According to the design prior and the proposed trial design, there was a 47% chance of a positive outcome (defined as a significant relative reduction in mortality of ≥15%), a 45% chance of a negative outcome (defined as a relative reduction in mortality <10%), and an 8% chance of ending in the consider zone (ie, a mortality reduction of 10 to <15%) for infants ≤1500 g (**Table 1**). For newborn infants ≤2000 g, we found a 37% chance of a positive outcome (defined as a significant relative reduction in mortality <10%), a 54% chance of a negative outcome, and a 9% chance of ending in the consider zone.

**Table 1:** Probability of study success in all-cause neonatal mortality for low birthweight newborn infants in Kenya*

Birthweight	Probabilities		
	**Go (%)**	**Consider (%)**	**Stop (%)**
Infants ≤1500 g	47%	8%	45%
Infants ≤2000 g	37%	9%	54%

*Success defined as observing a statistically significant relative reduction in mortality of at least 15% above standard of care.

## DISCUSSION

We applied the QDM framework to the NEWBORN study in order to calculate the probability that the study will demonstrate a significant reduction in neonatal mortality rates and therefore represent a sound investment from a financial and ethical standpoint. Elicitations of prior distribution, study simulations, and estimations of PoS were used to determine that the NEWBORN study, if it were to be conducted, would have a high probability of clearly answering its research questions. Assurance calculations derived a low (8%-9%) probability of the outcome ending in the consider zone, thus reinforcing the finding that the trial is likely to produce clear results.

Historically, before adopting prior elicitation and assurance simulations, GSK focused on power – a measure of the likelihood of observing a statistically significant result conditional upon some assumed but unknown value of the true treatment effect [17] – as the probability of statistical success in the context of major project milestones. However, a perceived inconsistency between GSK’s phase III trials being nominally powered at 90%, yet with lower actual success rates observed across the industry [30], pointed to power as an insubstantial measure for informing investment decisions. This led to the realisation that power statements are often misinterpreted as a substitute measure of PoS. Relying upon a power calculation as a proxy for PoS may thus jeopardise transparency in terms of the extent to which a trial has potential to achieve success [17].

The concepts of assurance and prior elicitation are becoming increasingly established in the literature as a broader and more relevant measure of PoS [17,31]. More widespread adaptation of prior elicitation and assurance is encouraged because their routine application provides several benefits. Compared to power, estimated PoS is more meaningful as a basis, as it captures current knowledge and uncertainties about treatment effect, thereby supporting discussions of trial design and objectives. Second, transparent insight is provided to the assumptions that are driving PoS, further refining study design and enhancing team understanding [19]. Also, the probability of observing clinically relevant effect sizes is explicitly characterised in advance. Moreover, the relationship between PoS and sample size can be assessed such that an optimal sample size can be identified in terms of incremental return on investment [17]. Finally, conventional statistical tests tend to dichotomise results according to whether they are or are not significant and do not allow decision makers to take explicit account of additional evidence – for example, of biological plausibility or of biases in the studies. In contrast, data presented as a series of posterior probability distributions better guide policy decisions by reflecting the reality that degrees of belief are often continuous, not dichotomous, and often vary from one person to another in the face of inconclusive evidence [32].

Thus, the use of assurance and prior elicitation has become routine within GSK as projects approach major milestones such as committing to phase III development. A part of the QDM framework at GSK consists of (1) determining a prior distribution for the effect(s) of interest used to support assurance calculations (eg, effect for treatment, effect for control, or effect for the difference between them) derived through various processes such as from individual expert opinions and/or a data-driven prior; and (2) an outline of the proposed study design and a clear definition of success in clinical and statistical terms so that assurance is aligned with specific target clinical profiles. Graphic visualisations of the relationship between assurance, study design and sample size may also be informative. Various other pharmaceutical companies have also adopted assurance to inform Go/No-Go decisions and aid trial design [31,33-36].

The use of assurance in tandem with prior elicitation can be integrated with other means of assessment and prioritisation of global health interventions, such as the Child Health and Nutrition Research Initiative (CHNRI) approach [21,37]. While CHNRI shares an element of prior distribution, QDM extends assessment of evidence to incorporate additional statistical calculations of study risk and its integration of various forms of prior elicitation, for example, including Delphi, the Sheffield Elicitation Framework, and others [38,39]. Given the limitations of bias that often appear in panel-based prior elicitation, it is important to ensure that a balance of external and internal experts are utilised as part of the prior elicitation process to counteract potential bias, guiding investors and those involved in study design to an accurate depiction of study risk and success.

The application of QDM to the NEWBORN study is one application of many in which evaluating assurance has played an important role in project decision-making or study design. Since 2017, project teams at GSK have routinely assessed assurance for projects and studies seeking endorsement by GSK governance committees. As a result of this assessment, there was a suggestion that the NEWBORN study had a high probability of producing clear results. Onset of the COVID-19 pandemic, however, delayed implementation of the study beyond the end-date for the GSK/Save the Children partnership strategy under which the study would have been implemented.

This study sheds light on the value of PPPHs in synthesizing disparate yet complementary skills and perspectives present in the private and public sectors. Through partnerships like that of GSK, Save the Children, and representatives from Stanford and Oxford Universities in collaboration with KEMRI, the reciprocal expertise of industry, NGOs, academia and government may bring industry stakeholders and those involved in study design closer to developing interventions of maximal benefit to beneficiaries. Benefits of approaching global health problems from a multisectoral perspective can come in the form of resources, intellectual input, contextual refinement, investment methodologies, and value assessment frameworks like QDM. Moreover, co-ownership of the process by NGO and governmental partners helps to ensure uptake of QDM findings into research and ultimately policy and programme implementation. Tools from the private sector can also be applied to global health independent of the pharmaceutical industry, and vice versa. For instance, a funder may use QDM to assess the PoS of a study, a researcher to refine their study design, a grant writer to provide a quantitative basis for their proposal, or an NGO, government or in-country partner to decide whether to allocate resources to a program. If adopted on a global scale, such partnerships may thus accelerate the advancement of global health.

### Limitations

Despite its strengths, the QDM framework possesses limitations. While QDM integrates uncertainty about the treatment effect, if bias is present in the prior elicitation stage, the assurance calculation may be affected. This shortcoming can be met by maintaining transparency when communicating assurance to decision makers by including how experts arrived at a specific prior as well as a summary of the prior alongside presentation of assurance values and where applicable highlighting any potential conflict of interest among member of the prior elicitation panel. As applied to the NEWBORN study in particular, for instance, a potential conflict of interest in promoting the study might have existed for five out of nine of the panellists, who were involved in the NEWBORN study design, two of whom were GSK employees, as noted in Table S1 in the **Online Supplementary Document**. Four of nine of the panel members were GSK employees; however, the QDM analysis was performed under an explicitly non-profit global health program, and there was no potential financial conflict of interest. Finally, the involvement of a limited group of technical experts and stakeholders in a prior elicitation panel may lead to bias. To mitigate potential bias, however, expert panel members must provide a written justification of their beliefs, referencing prior research. While the expert panel was small, we sought to ensure diverse representation, which appears to be reflected in the members’ broad ranges of certainty in levels of reduction in mortality (**Figure 2**). This level of uncertainty was used to construct the prior distribution which was then used to model data for possible outcomes. Hence, the variability in the views of the panel members – many of whom indicated the reduction in mortality could lie below or above the 15% clinical cut-off – was captured in the simulations which derived a low probability of landing in the consider zone (8%-9%), which provides reassurance that if the study was conducted, a clear answer to the research question likely would be obtained.

## CONCLUSIONS

At the cutting edge in industry applied to global health, QDM is part of a rigorous assessment process of whether to make an investment in an intervention by quantifying the risks and PoS associated with a given study, for the benefit of patients/subjects, investors and researchers. QDM is a promising method of calculating study PoS that could be used alongside other research and investment prioritisation tools in global health. The NEWBORN study is a case study of a PPPH bringing together multiple partners representing NGO, academia, industry and governmental sector to inform the viability of a study as an investment and to create co-ownership for research design. Investigating the PoS of a study using QDM maximises the efficiency and benefit of research to diverse stakeholders in global health.

## Additional material


Online Supplementary Document

## References

[1] Kostyak L Shaw DM Elger B Annaheim B A means of improving public health in low- and middle-income countries? Benefits and challenges of international public-private partnerships. Public Health. 2017;149:120-9. 10.1016/j.puhe.2017.03.005 28595064

[2] Hodge G. Pharmaceuticals and the poor. 2013. Available: http://www.ethical-goods.com/gsk/. Accessed: 14 January 2022.

[3] WEF. Development-driven public-private partnerships in health, emerging priorities from roundtable discussions. 2005. Available: http://www.weforum.org/pdf/ppp_health_summary.pdf. Accessed: 14 January 2022.

[4] Marsh DR Darmstadt GL Moore J Daly P Oot D Tinker A Advancing newborn health and survival in developing countries: a conceptual framework. J Perinatol. 2002;22:572-6. 10.1038/sj.jp.7210793 12368975

[5] Darmstadt GL Saha SK Ahmed AS Chowdhury MAKA Law PA Ahmed S Effect of topical treatment with skin barrier-enhancing emollients on nosocomial infections in preterm infants in Bangladesh: a randomised controlled trial. Lancet. 2005;365:1039-45. 10.1016/S0140-6736(05)71140-5 15781099

[6] Yoshio H Tollin M Gudmundsson G Antimicrobial polypeptides of human vernix caseosa and amniotic fluid: Implications for newborn innate defense. Pediatr Res. 2003;53:211-6. 10.1203/00006450-200302000-00003 12538777

[7] Hammarlund K Sedin G Transepidermal water loss in newborn infants, III: relation to gestational age. Acta Paediatr Scand. 1979;68:795-801. 10.1111/j.1651-2227.1979.tb08214.x 539401

[8] Bhutta Z Neonatal bacterial infections in developing countries: strategies for prevention. Semin Neonatol. 1999;4:159-71. 10.1016/S1084-2756(99)90040-4

[9] Darmstadt GL The skin and nutritional disorders of the newborn. Eur J Pediatr Dermatol. 1998;8:221-8.

[10] Irimu G Aluvaala J Malla L Omoke S Ogero M Mbevi G Neonatal mortality in Kenyan Hospitals: a multi-site, retrospective, cohort study. BMJ Glob Health. 2021;6:e004475. 10.1136/bmjgh-2020-004475 34059493 PMC8169483

[11] Aluvaala J Collins GS Maina B Mutinda C Wayiego M Berkley JA Competing risk survival analysis of time to in-hospital death or discharge in a large urban neonatal unit in Kenya. Wellcome Open Res. 2019;4:96. 10.12688/wellcomeopenres.15302.1 31289756 PMC6611136

[12] Were FN Bwibo NO Neonatal nutrition and later outcomes of very low birthweight infants at Kenyatta national hospital. Afr Health Sci. 2007;7:108-14. 17594288 10.5555/afhs.2007.7.2.108 PMC1925270

[13] Were FN Bwibo NO The contribution of very low birth weight deaths to infant mortality. East Afr Med J. 2009;86:374-7. 20575311 10.4314/eamj.v86i8.54157

[14] Salam RA Das JK Darmstadt GL Bhutta ZA Emollient therapy for preterm newborn infants – evidence from the developing world. BMC Public Health. 2013;13 Suppl 3:S31. 10.1186/1471-2458-13-S3-S31 24564550 PMC3878124

[15] Jabraeile M Rasooly AS Farshi MR Malakouti J Effect of olive oil massage on weight gain in preterm infants: A randomized controlled clinical trial. Niger Med J. 2016;57:160-3. 10.4103/0300-1652.184060 27397955 PMC4924397

[16] O’Hagan A Stevens JW Bayesian assessment of sample size for clinical trials of cost-effectiveness. Med Decis Making. 2001;21:219-30. 10.1177/02729890122062514 11386629

[17] Crisp A Miller S Thompson D Best N Practical experiences of adopting assurance as a quantitative framework to support decision making in drug development. Pharm Stat. 2018;17:317-28. 10.1002/pst.1856 29635777

[18] O’Hagan A Stevens JW Campbell MJ Assurance in clinical trial design. Pharm Stat. 2005;4:187-201. 10.1002/pst.175

[19] Dallow N Best N Montague T Better decision making in drug development through adoption of prior elicitation. Pharm Stat. 2018;17:301-16. 10.1002/pst.1854 29603614

[20] Garthwaite PH Kadane JB O’Hagan A Statistical methods for eliciting prior distributions. J Am Stat Assoc. 2005;100:680-701. 10.1198/016214505000000105

[21] Rudan I Yoshida S Chan KY Sridhar D Wazny K Nair H Setting health research priorities using the CHNRI method: VII. A review of the first 50 applications of the CHNRI method. J Glob Health. 2017;7:011004. 10.7189/jogh.07.011004 28685049 PMC5481891

[22] Aluvaala J Collins G Maina B Mutinda C Waiyego M Berkley JA Prediction modelling of inpatient neonatal mortality in high-mortality settings. Arch Dis Child. 2020;106:449-54. 10.1136/archdischild-2020-319217 33093041 PMC8070601

[23] World Health Organization. WHO recommendations on interventions to improve preterm birth outcomes, 2015. Available: https://pubmed.ncbi.nlm.nih.gov/26447264/. Accessed 18 April 2022. 26447264

[24] World Health Organization. Optimal feeding of low birth weight infants in low and middle income countries, 2011. Available: https://apps.who.int/iris/bitstream/handle/10665/85670/9789241548366_eng.pdf?sequence=1&isAllowed=y&ua=1. Accessed 24 March 2022. 26042325

[25] Aluvaala J Okello D Murithi G Wafula L Wanjala L Isika N Delivery outcomes and patterns of morbidity and mortality for neonatal admissions in five Kenyan hospitals. J Trop Pediatr. 2015;61:255-9. 10.1093/tropej/fmv024 25841436 PMC4514903

[26] English M Gathara D Nzinga J Kumar P Were F Warfa O Lessons from a Health Policy and Systems Research programme exploring the quality and coverage of newborn care in Kenya. BMJ Glob Health. 2020;5:e001937. 10.1136/bmjgh-2019-001937 32133169 PMC7042598

[27] Murphy GAV Gathara D Mwachiro J Abuya N Aluvaala J English M Effective coverage of essential inpatient care for small and sick newborns in a high mortality urban setting: a cross-sectional study in Nairobi City County, Kenya. BMC Med. 2018;16:72. 10.1186/s12916-018-1056-0 29783977 PMC5963150

[28] Ahmed AS Saha SK Chowdhury MA Law PA Black RE Santosham M Acceptability of massage with skin barrier-enhancing emollients in young neonates in Bangladesh. J Health Popul Nutr. 2007;25:236-40. 17985826 PMC2754003

[29] LeFevre A Shillcutt SD Saha SK Ahmed ASMNU Ahmed S Chowdhury MAKA Cost-effectiveness of skin-barrier-enhancing emollients among preterm infants in Bangladesh. Bull World Health Organ. 2010;88:104-12. 10.2471/BLT.08.058230 20428367 PMC2814477

[30] Biotechnology Innovation Organisation. Clinical development success rates 2005–2016. 2015. Available: https://www.bio.org/ sites/default/files/Clinical%20Development%20Success%20Rates%202006-2015%20-%20BIO,%20Biomedtracker,%20Amplion%202016.pdf. Accessed: 3 December 2017.

[31] Hong S Shi L Predictive power to assist phase 3 go/no go decision based on phase 2 data on a different endpoint. Stat Med. 2012;31:831-43. 10.1002/sim.4476 22302442

[32] Lilford RJ Braunholtz D The statistical basis of public policy: a paradigm shift is overdue. BMJ. 1996;313:603-7. 10.1136/bmj.313.7057.603 8806254 PMC2352073

[33] Sabin T Matcham J Bray S Copas A Parmar MK A quantitative process for enhancing end of phase 2 decisions. Stat Biopharm Res. 2014;6:67-77. 10.1080/19466315.2013.852617 24683441 PMC3967501

[34] Wang Y Fu H Kulkarni P Kaiser C Evaluating and utilizing probability of study success in clinical development. Clin Trials. 2013;10:407-13. 10.1177/1740774513478229 23471634

[35] Walley RJ Smith CL Gale JD Woodward P Advantages of a wholly Bayesian approach to assessing efficacy in early drug development: a case study. Pharm Stat. 2015;14:205-15. 10.1002/pst.1675 25865949

[36] Jiang K Optimal sample sizes and go/no-go decisions for phase II/III development programs based on probability of success. Stat Biopharm Res. 2011;3:463-75. 10.1198/sbr.2011.10068

[37] Rudan I Gibson J Kapiriri L Lansang MA Hyder AA Lawn J Setting priorities in global child health research investments: assessment of principles and practice. Croat Med J. 2007;48:595-604. 17948946 PMC2205967

[38] Linstone HA, Turoff M, editors. The Delphi Method: Techniques and Applications. Massachusetts: Addison-Wesley; 1975.

[39] Dolan JG Veazie PJ Harnessing expert judgment to support clinical decisions when the evidence base Is weak. Med Decis Making. 2019;39:74-9. 10.1177/0272989X18810178 30517823

