# Supplementary material for: Quantitative decision making for investment in global health intervention trials: Case study of the NEWBORN study on emollient therapy in preterm infants in Kenya
Source: J Glob Health. 2022 Jun 11;12:04045. doi: 10.7189/jogh.12.04045 (PMC9185187; doi:10.7189/jogh.12.04045)
Supplement: Online Supplementary Document [file jogh-12-04045-s001.pdf]

Appendix Table S1: Prior elicitation panel members

Panel Expert	Affiliation
Gary Darmstadt*	Associate Dean for Maternal and Child Health, and Professor of Neonatal and Developmental Paedeatrics in the Department of Pedeatrics at the Stanford University School of Medicine
Mike English*	Senior Research Fellow at *KEMRI*-Wellcome Trust. Professor, Oxford University
Sindura Ganapathi	Deputy Director, Global Health Program, Bill & Melinda Gates Foundation<br>Current position: Visiting PSA Fellow, Office of the Principal Scientific Advisor, Government of India
Sarah Williams*	Senior Maternal and Newborn Health Advisor, Save The Children
Kathleen Beach	Vice President, Early Development Program, Biocryst<br>Current position: Independent consultant
Rachel Gibson*	Clinical Development Director, Global Health, GSK
Annie Stylianou*	Statistical Lead, Global Health Catalyst, GSK
Christopher Were	Country Medical Director – Kenya, GSK
Naveen Sankar	Cluster Medical Director / Africa Cluster / Export Markets, GSK
*involved in study design (potential conflict of interest)	

Table S2. Summary of publications presented to prior elicitation panel

Publication	Description	Location	Sample size	Interventions	Effect Sizes	Conclusions/findings	Limitations
Cleminson J, McGuire W. Topical emollient for preventing infection in preterm infants. Cochrane Database Syst Rev. 2016 Jan 29;2016(1):CD001150. doi: 10.1002/14651858.CD001150.pub3.	Cochrane Review addressing the question "Does the topical application of emollients reduce the incidence of invasive infection in preterm infants?"	Most participants were very preterm infants cared for in health-care facilities in high-income countries.	3089 infants 18 eligible primary publications (21 trial reports)	Eleven trials (1184 infants) assessed the effect of sunflower, sunflower seed, and other vegetable oils. Nine of these trials were undertaken in low- or middle-income countries and all were based in health-care facilities rather than home or community settings.	Subgroup of trials conducted in low- and middle-income countries: RR for prevention of invasive infection: 0.71 (0.51 to 1.01); RR for mortality: 0.94 (0.81 to 1.08).	Analyses of these trial data provided low quality evidence that emollients prevent infection in preterm infants but not mortality.<br><br>Since these interventions are low cost, readily accessible and generally acceptable, further randomised controlled trials, particularly in both community- and health care facility-based settings in low-income countries, may be justified.	Most participants were very preterm infants cared for in health-care facilities in high-income countries. The epidemiology of invasive infection in preterm or low birth weight infants in low- or middle-income countries differs from that in high-income countries (Zaidi 2005). The overall incidence in low- or middle-income countries is much higher but infections are less likely to be directly associated with intensive care or invasive procedures. Most infections are due to Gram-negative bacilli and the attributable mortality and morbidity is greater than in high-income settings.
Darmstadt GL, Saha SK, Ahmed AS, Chowdhury MA, Law PA, Ahmed S, Alam MA, Black RE, Santosham M. Effect of topical treatment with skin barrier-enhancing emollients on nosocomial infections in	A RCT to ascertain whether application of emollients to enhance skin barrier function would prevent nosocomial infections in this population (<33 weeks) in Bangladesh<br><br>Approx 300 neonates randomised to daily massage with sunflower seed oil (n=159) or Aquaphor	Bangladesh	497 infants	Sunflower seed oil (n=159) or Aquaphor (petrolatum, mineral oil, mineral wax, lanolin alcohol; n=157)	Adjusted incidence rate ratio [IRR] 0.59, 95% CI 0.37-0.96, p=0.032	Overall, infants treated with sunflower seed oil were 41% less likely to develop nosocomial infections than controls. Aquaphor did not significantly reduce the risk of infection.<br><br>Findings confirm that skin application of sunflower seed oil provides protection against nosocomial infections in preterm very low birthweight infants. The low cost, availability, simplicity, and effect of treatment make it an important intervention for very low birthweight infants admitted to hospital in developing countries.	Study had insufficient power to assess the overall effect of Aquaphor therapy on incidence of nosocomial infections.

preterm infants in Bangladesh: a randomised controlled trial. Lancet. 2005 Mar 19-25;365(9464):1039-45. doi: 10.1016/S0140-6736(05)71140-5. PMID: 15781099.	(petrolatum, mineral oil, mineral wax, lanolin alcohol; n=157) to look at bloodstream proven infections						
Darmstadt GL, Saha SK, Ahmed AS, Choi Y, Chowdhury MA, Islam M, Law PA, Ahmed S. Effect of topical emollient treatment of preterm neonates in Bangladesh on invasion of pathogens into the bloodstream. Pediatr Res. 2007 May;61(5 Pt 1):588-93. doi: 10.1203/pdr.0b013e3180459f75. PMID: 17413870.	Paper examines skin and blood flora to gain insights into barrier function in preventing infection	Bangladesh	496 infants	Sunflower seed oil (n = 159), or Aquaphor (petrolatum, mineral oil, mineral wax, lanolin alcohol (n = 156)	Skin condition scores at 3 d were better in patients treated with either emollient compared with untreated controls; however, skin flora was similar across the groups. The SSO group showed a 72% elevated odds of having a false-positive (FP) skin culture associated with a negative blood culture (i.e. skin flora was blocked at the skin surface from entering into the blood) compared with the control group.	Topical therapy with SSO reduced the passage of pathogens from the skin surface into the bloodstream of preterm infants.	Unable to measure transepidermal water loss in the study setting, and thus direct, definitive data on skin barrier function was lacking in this study. However, the data on skin condition suggests that skin barrier function was improved in infants treated with SSO.

Darmstadt GL, Ahmed S, Ahmed AS, Saha SK. Mechanism for prevention of infection in preterm neonates by topical emollients: a randomized, controlled clinical trial. Pediatr Infect Dis J. 2014 Nov;33(11):1124-7. doi: 10.1097/INF.0000000000000423. PMID: 24853544.	Paper looks at the importance of early initiation of treatment	Bangladesh	491 infants	High-linoleate SSO (n = 154) (Omega Nutrition, Bellingham, WA), and Aquaphor (n = 159) (petroleum, mineral oil, mineral wax and lanolin alcohol) (Beiersdorf, Norwalk, CT).	Rate of deterioration of skin condition was significantly lower (P < 0.05) in both emollient arms compared with the untreated control group. Emollients reduced the incidence of infection only when the skin had no signs of deterioration.<br><br>Adjusted odds ratio of skin score for infection was 1.32 (95% confidence interval: 1.06-1.65). Aquaphor incidence rate ratio: 0.43 (95% confidence interval: 0.19-0.97) Sunflower seed oil incidence rate ratio: 0.46 (95% confidence interval: 0.21-0.99).	(1) Skin score increased over time during the neonatal period, indicative of deterioration of skin integrity; (2) emollient therapy significantly reduced the rise in skin score, reflecting protection from skin injury; (3) risk of bloodstream infection increased significantly with compromise in skin integrity; (4) the protective effect of emollient therapy with regard to infection was seen only in the absence of skin injury (when the skin score is 0) and (5) results were similar for an emollient that is inert in the skin (Aquaphor) and one that is metabolically active (SSO). These observations support the hypothesis that emollients act by preserving skin integrity. Furthermore, these data suggest that once skin injury occurs emollients do little to protect from pathogens gaining access to portals of entry in the skin and to the bloodstream to cause invasive infections. Thus, these findings highlight the importance of early initiation and regular applications of emollients to maintain skin integrity and protect the skin from injury.	Results suggest that a direct measure of skin injury may be more predictive of the ability of an emollient to provide protection from infection. This study used a crude visual measure of skin condition; innovation in measures of skin integrity are needed to define biomarkers which more accurately predict the ability of emollients to improve human health outcomes such as serious infections.
Salam RA, Darmstadt GL, Bhutta ZA. Effect of emollient therapy on clinical outcomes in preterm neonates in Pakistan: a	A RCT to assess efficacy of coconut oil in a cohort (n=258) of hospital born preterm infants<br>Preterm infants >26 and < 37 weeks and weighing >750g<br>Conducted in Pakistan conducted	Pakistan	258	Coconut oil (n=128)	Unadjusted hazard for developing hospital-acquired infection in the control group was 4.7 (95% CI 1.8 to 12.4) compared with the intervention group. Hazard for hospital-acquired infection in the control group was 6.0 (95% CI 2.3 to 16)	Topical emollient therapy was effective in maintaining skin integrity and reducing the risk of bloodstream infection in preterm infants in a tertiary hospital setting in Pakistan.	Appropriate placebo to compare the effect of CO to could not be developed, the physicians and nurses could not be blinded to administration of emollient as they were present in the NICU and nursery around-the-clock and the loss to follow-up was higher than expected (10%).

randomised controlled trial. Arch Dis Child Fetal Neonatal Ed. 2015 May;100(3): F210-5. doi: 10.1136/archdischild-2014-307157. Epub 2015 Jan 30. PMID: 25637007.	between 2011-2012.				compared with the intervention group. Mean weight gain was 11.3 g/day higher (95% CI 8.1 to 14.6, p<0.0001)		
Salam RA, Das JK, Darmstadt GL, Bhutta ZA. Emollient therapy for preterm newborn infants--evidence from the developing world. BMC Public Health. 2013;13 Suppl 3(Suppl 3):S31. doi: 10.1186/1471-2458-13-S3-S31. Epub 2013 Dec 20. PMID: 24564550;	A systematic review of literature (published up to December 2012) describing the effectiveness of emollient therapy.	LMIC countries	7 studies and 1 unpublished trial	Sunflower, coconut, soybean or mineral oil	Topical emollient therapy significantly reduced neonatal mortality by 27% (RR: 0.73, 95% CI: 0.56, 0.94) and hospital acquired infection by 50% (RR: 0.50, 95% CI: 0.36, 0.71). There were significant increases in weight (g) (MD: 98.04, 95% CI: 42.64, 153.45) and weight gain (g/kg/day) (MD: 1.57, 95% CI: 0.79, 2.36), whereas the impacts were non-significant for length and head circumference.	Emollient therapy is associated with improved weight gain, reduced risk of infection and associated newborn mortality in preterm neonates and is a potentially promising intervention for use in low resource settings. Large scale effectiveness trials are required to further assess the impact of this intervention.	Had data on mortality but since the evidence was weak, we used hospital acquired infection (severe morbidity) as a proxy for neonatal mortality and propose that topical emollient therapy for preterm neonates can reduce neonatal infection related mortality by 50% among preterm neonates <37 weeks gestation.

PMCID: PMC3878124.							
Darmstadt GL, Saha SK, Ahmed AS, Ahmed S, Chowdhury MA, Law PA, Rosenberg RE, Black RE, Santosham M. Effect of skin barrier therapy on neonatal mortality rates in preterm infants in Bangladesh: a randomized, controlled, clinical trial. Pediatrics. 2008 Mar;121(3):522-9. doi: 10.1542/peds.2007-0213. PMID: 18310201.	A RCT to evaluate topical application of emollients to improve survival rates among hospitalized preterm infants (<33 weeks)	Bangladesh	497	Sunflower seed oil (n= 159) or Aquaphor (petrolatum, mineral oil, mineral wax, lanolin alcohol; n= 157)	The overall mortality rate among all patients enrolled in the trial was 64%. The neonatal mortality rate was significantly reduced (P = .042), by 26%, in infants treated with SSO (hazard-adjusted ratio: 0.74; 95% CI: 0.55–0.99). Treatment with Aquaphor also significantly reduced mortality rates, by 32% (hazard-adjusted ratio: 0.67; 95% CI: 0.51–0.92; P = .013). In very preterm infants (gestational age of <32 weeks; n = 371), mortality rates were reduced by 31% (95% CI: 6%–50%; P = .021) and 38% (95% CI: 13%–55%; P = .005) by SSO and Aquaphor treatment, respectively.	Treatment with sunflower seed oil resulted in a statistically significant 26% reduction in mortality rates, compared with infants not receiving topical emollient therapy. Aquaphor therapy also significantly reduced mortality rates, by 32%.<br><br>Topical therapy with skin barrier-enhancing emollients improved survival rates among preterm hospitalized infants in Bangladesh. This study provides evidence for the implementation of topical therapy for high-risk preterm neonates in developing countries.	This study had sufficient but limited power to detect differences in neonatal mortality rates among treatment groups. Further investigation of this intervention is warranted, including phase 4 studies to evaluate the implementation of this intervention in several developing-country hospitals.
Pupala SS, Rao S, Strunk T, Patole S. Topical application of coconut oil to the skin	Systemic review of coconut oil Majority of trials. Included normal term infants.	LMIC countries	727 infants 7 trials	Coconut oil or control	Meta-analysis using random effects model found significantly lower incidence of hospital-acquired blood stream infections (HABSI) in the coconut oil group	Overall, infants in the coconut oil group had decreased water loss, decreased infection rates, better growth and skin condition. There were no significant adverse effects associated with coconut oil application.	The overall quality of evidence was considered moderate for the outcome of HABSI and low for the outcome of physical growth based on GRADE guidelines.

of preterm infants: a systematic review. Eur J Pediatr. 2019 Sep;178(9):1317-1324. doi: 10.1007/s00431-019-03407-7. Epub 2019 Jul 2. PMID: 31267223.					(11/164 vs 32/166; relative risk 0.35, 95% confidence interval 0.18, 0.67, p = 0.001; I2 = 0%, two RCTs).	Topical application of coconut oil may reduce the risk of infection and improve weight gain and skin condition in preterm infants. However, the quality of evidence was considered to be moderate to low based on GRADE guidelines.	
Kusari A, Han AM, Virgen CA, Matiz C, Rasmussen M, Friedlander SF, Eichenfield DZ. Evidence-based skin care in preterm infants. Pediatr Dermatol. 2019 Jan;36(1):16-23. doi: 10.1111/pde.13725. Epub 2018 Dec 12. PMID: 30548578.	Evidence-based review of the literature on skin care of preterm neonates<br>This paper talks to skin development / gestational age and weight – 1.5kg – 32 weeks.	Papers regarding the use of emollients on premature infant skin care in developing countries took place in LMIC settings	68 articles	General skincare in preterm neonates general skincare in preterm neonates, inluding emollients and massage (Sunflower seed oil, Aquaphor, coconut oil, mustard oil)	N/A	Topical emollients, particularly sunflower seed oil, appear to reduce the incidence of skin infections in premature neonates—but only in developing countries. In developed countries, studies suggest that topical petrolatum ointment may increase the risk of candidemia and coagulase-negative Staphylococcus infection in the preterm population, perhaps by creating a milieu similar to occlusive dressings. For preterm infants with catheters, povidone-iodine and chlorhexidine are comparably effective at preventing catheter colonization.<br><br>Compared to term infants, preterm infants possess fragile skin barriers and are particularly susceptible to environmental fluctuations and infectious assault, and thus are in greater need of appropriate prophylactic and therapeutic interventions.<br><br>Highlight the need for high-quality research in this area.	None outlined.

Nangia S, Paul VK, Deorari AK, Sreenivas V, Agarwal R, Chawla D. Topical Oil Application and Trans-Epidermal Water Loss in Preterm Very Low Birth Weight Infants-A Randomized Trial. J Trop Pediatr. 2015 Dec;61(6):414-20. doi: 10.1093/tropej/fmv049. Epub 2015 Sep 3. PMID: 26338490.	Randomized trial in very low birth weight (VLBW) neonates (750-1500g) conducted in 2006. Seventy-four neonates randomised to receive coconut oil (without massage) or standard of care Study looked at whether TEWL was reduced in the Oil group Lower TEWL in the Oil group indicates protective effect of oil on the skin and is believed to be key to optimal management and improved outcome	India	74 infants	Coconut oil	Birth weight (g; mean ± SD: 1213 + 214 vs. 1164 + 208, p = 0.31), gestation [week; median (interquartile range): 32 (31–33) vs. 32 (29–33), p = 0.10] and other baseline variables were comparable. TEWL was significantly reduced (g/m 2 /h, mean difference: −6.80, 95% confidence interval: −3.48, −10.15; p < 0.01) with better skin condition and lower bacterial growth in the Oil group (20% vs. 60%, p < 0.01).	Coconut oil application accelerates the physiological decline in TEWL during first week of life, reduces skin colonization and results in a better skin condition.	Small sample size.
Kanti V, Grande C, Stroux A, Bührer C, Blume-Peytavi U, Garcia Bartels N. Influence of sunflower seed oil on the skin barrier function of preterm infants: a randomized controlled	RCT to investigate the effect of sunflower seed oil on skin barrier development in low-birth-weight premature infants <37 weeks gestation(1500-2500g). Twenty-two neonates randomized to oil or control group for 10 days postnatal. The primary outcome was Trans-Epidermal	Berlin	22 low-birth-weight (1500-2500g) and premature infants <37 weeks gestation	Sunflower seed oil (n=11) or control (n=11)	Original sample size calculation was based on an effect size of 1.0 (i.e. a difference in means and a common standard deviation, SD, of 2), a power of 80% and a 2-sided significance level of 0.05. Due to the recruitment difficulties in this age group, 11 infants per group could be included by the end of the study period, giving an effect size of 1.3, i.e. the SD being	Skin pH decreased, while sebum remained stable in both groups. In group C, TEWL remained stable; in group SSO, TEWL increased significantly on the abdomen, leg and buttock until day 11, followed by a decrease after SSO application had been stopped. Abdomen SCH remained stable in group C, but continuously decreased in group SSO until day 21.<br><br>SSO application may retard postnatal skin barrier maturation in preterm infants.	Small sample size.

trial. Dermatology. 2014;229(3): 230-9. doi: 10.1159/000363380. Epub 2014 Oct 15. PMID: 25323538.	Water Loss (TEWL)				1.3-fold the difference between study arms.		
Darmstadt GL, Badrawi N, Law PA, Ahmed S, Bashir M, Iskander I, Al Said D, El Kholy A, Husein MH, Alam A, Winch PJ, Gipson R, Santosham M. Topically applied sunflower seed oil prevents invasive bacterial infections in preterm infants in Egypt: a randomized, controlled clinical trial. Pediatr Infect Dis J. 2004 Aug;23(8):719-25. doi: 10.1097/01.inf.00001330	RCT to assess impact of sunflower seed oil on rates of nosocomial infection and mortality in preterm infants <34 weeks gestational.	Egypt	103 preterm infants <34 weeks gestational	Sunflower seed oil (n = 51) and topical prophylaxis (n = 52)	Sunflower seed oil resulted in a significant improvement in skin condition (P = 0.037) and a highly significant reduction in the incidence of nosocomial infections (adjusted incidence ratio, 0.46; 95% confidence interval, 0.26-0.81; P = 0.007) compared with infants not receiving topical prophylaxis. There were no reported adverse events as a result of topical therapy.	Topical therapy with SSO was safe and highly efficacious in improving skin condition and reducing the incidence of nosocomial infections. Treatment with sunflower seed oil resulted in a significant improvement in skin condition and a highly significant reduction in the incidence of nosocomial infections compared with infants not receiving topical prophylaxis. There were no reported adverse events as a result of topical therapy.<br><br>Given the low cost (~$.20 for a course of therapy) and technologic simplicity of the intervention and the effect size observed in this study, a clinical trial with increased numbers of subjects is indicated to evaluate the potential of topical therapy to reduce infections and save newborn lives in developing countries.	Power of the study limited due to high mortality rate. More than 60% of the patients died (64 of 103), curtailing the number of surveillance days available for observing an effect of the emollient.

47.50836.6f. PMID: 15295221.							
Darmstadt GL, Mao-Qiang M, Chi E, Saha SK, Ziboh VA, Black RE, Santosham M, Elias PM. Impact of topical oils on the skin barrier: possible implications for neonatal health in developing countries. Acta Paediatr. 2002;91(5):546-54. doi: 10.1080/080352502753711678. PMID: 12113324.	Study investigated the epidermal barrier-enhancing effects of various vegetable oils in 6 to 8 wk old male, hairless mice	USA	6 to 8 wk-oldm ale, hairless mice (Hr/Hr, Charles River, Cambridge ,M A)	Sunflower seed oil (n=12), mustard oil (n=11), olive oil (n=12), soybean oil (n=10), aquaphor (n=22)	N/A	A single application of sunflower seed oil significantly accelerated skin barrier recovery within 1 h; the effect was sustained 5 h after application. In contrast, the other vegetable oils tested (mustard, olive and soybean oils) all significantly delayed recovery of barrier function compared with control- or Aquaphor-treated skin. Twice-daily applications of mustard oil for 7 d resulted in sustained delay of barrier recovery. Moreover, adverse ultrastructural changes were seen under transmission electron microscopy in keratin intermediate filament, mitochondrial, nuclear, and nuclear envelope structure following a single application of mustard oil.	Caution must be exercised in extrapolating to the human neonate based on results from the tape-stripped hairless mouse model.
Topical Emollient therapy for promotion of newborn health and survival	Summary from Professor Gary Darmstadt on a recent India community-based emollient trial.	India	13,478 and 13,109 newborns	Sunflower seed oil (n=4,096), mustard oil (n=4,720)	Intervention infants gained significantly more weight by 0.95 g/kg/day (95% CI 0.07 1.82, p=0.03) than comparison infants in intention-to-treat analysis and by 1.31 g/kg/d (95% CI 0.17-2.46, p<0.02) in per protocol analysis. Restricted cubic spline regression revealed the largest differences in	SSO improved neonatal growth at population-level, especially in very low birth weight infants, when used across the facility-community continuum of care. Given low adherence, further research is needed to develop innovative approaches to improving demand for recommended therapy inside hospital as well as in community settings.	It was not possible to mask study arm allocation to study workers or participants. Thus, it is possible that care provided to SSO treated infants differed from comparison infants in ways that could not be accounted for. However, researchers took a number of measures throughout the trial to minimize bias and potential Hawthorne effect. Per-protocol analysis may be subject to bias. Measurement of emollient use relied on

					weight gain – 2-3 g/kg/day – occurred in infants <2000 g. No group differences in morbidities were found by intention-to-treat analysis but in per-protocol analysis, rates of hospitalization and of any illness were reduced by 36% [adjusted OR (aOR) 0.64 (95% CI 0.44-0.94), p=0.022] and 44% [aOR 0.56 (95% CI 0.40-0.77), p<0.001], respectively, in treated infants.		retrospective interviews from caregivers and not actual observation of oil application, and therefore, may be subject to respondent bias. It is also possible that families which adhered to recommended emollient practices may be more likely to adhere to general recommendations on newborn care by other health workers such as governmental frontline workers, thus potentially over-estimating the actual impact of the intervention.<br><br>Adherence to recommended emollient therapy practices was also limited.

Appendix Table S3A: Simulations for bi-modal design prior for infants ≤1500 g

```
*******************************************************************************;
* Prior Elicitation: Probability that emollient has no benefit above SoC is 34% for babies ≤1.5kg *;
* Simulate values between 0 to 1 and assign to normal distribution if *;
* value≤0.34 (no effect) or uniform distribution if value>0.34 (has effect) *;
*******************************************************************************;
data weighted_1500g;
do i=1 to 1000000;
      placebo = 0.5;      *assumes mortality rate for SoC is 50%;
      a=rand('uniform',0,1);
      if a LE 0.34 then prior=rand('normal',0.5,0.005);
      else prior=rand('uniform',0.25,0.50);  *1-50% relative reduction equates to an absolute of 0.25-0.50 based on SoC mortality of 50%;
output;
end;
run;
data save_sim.Prior_1500g; set weighted_1500g; run;
```

Appendix Table S3B: Study trial simulations for infants ≤1500 g

```
*********************************************************************;
* Assurance calculations for 'Go' decision if neonatal mortality reduction >=15%      *;
* and assurance calculations for 'Consider' decision if reduction is 10-15%               *;
*********************************************************************;
data assurance_1500g;
 set weighted_1500g;;
    Diff=placebo-prior;
           n = 526;           * n PER ARM;

      p1 = placebo;
      p2 = prior;
      pbar = (p1+p2)/2;
      se = sqrt(2*(pbar*(1-pbar)/n));
      z_alpha = probit(0.5);

      z_den=SQRT((p1*(1-p1))+(p2*(1-p2)));

      z_num_15=(SQRT(n))*(p1-p2-0.075);  * 15% relative reduction (of 0.5) equates to 0.075;

      z_15=(z_num_15/z_den)-z_alpha;

*******************************************************************************;
*The PROBNORM function returns the probability that an observation from the standard normal   *;
* distribution is less than or equal to x                                                                              *;
*******************************************************************************;
        power_15_diff=probnorm(z_15);

      z_num_10=(SQRT(n))*(p1-p2-0.05);    * 10% relative reduction (of 0.5) equates to 0.05;

      z_10=(z_num_10/z_den)-z_alpha;

*******************************************************************************;
*The PROBNORM function returns the probability that an observation from the standard normal *;
*distribution is less than or equal to x                                                                              *;
*******************************************************************************;
        power_10_diff=probnorm(z_10);

        assurance_go=power_15_diff;

        assurance_consider=power_10_diff-power_15_diff;

run;

PROC means data=assurance_1500g n mean;
TITLE1 'Assurance for babies <=1500g';
TITLE2 'Assuming 66% probability that emollient will have an effect above
SoC';
TITLE3 'Emollient effect believed to result in a relative reduction of 1-
50%';
TITLE4 'Uniform distribution used to fit effect curve and Normal distribution
to fit no effect curve';
      VAR PRIOR p1 p2 assurance_go assurance_consider;
RUN;
```

Appendix Table S3C: Output for study trial simulations for infants ≤1500 g

Assurance for babies <=1500g
Assuming 66% probability that emollient will have an effect above SoC
Emollient effect believed to result in a relative reduction of 1-50%
Uniform distribution used to fit effect curve and Normal distribution to fit no effect curve

The MEANS Procedure

Variable	N	Mean
prior	1000000	0.4158107
p1	1000000	0.5000000
p2	1000000	0.4158107
assurance_go	1000000	0.4743413
assurance_consider	1000000	0.0805216

Appendix Table S4A: Simulations for bi-modal design prior for infants ≤2000 g

```
*********************************************************************************;
* Prior Elicitation: Probability that emollient has no benefit above SoC is 52% for babies ≤2kg          *;
* Simulate values between 0 to 1 and assign to normal distribution if                                   *;
* value≤0.52 (no effect) or uniform distribution if value>0.52 (has effect)                              *;
*********************************************************************************;
data weighted_2000g;
do i=1 to 1000000;
      placebo = 0.3;      *assumes mortality rate for SoC is 30%;
      a=rand('uniform',0,1);
      if a LE 0.52 then prior=rand('normal',0.3,0.005);
      else prior=rand('uniform',0.15,0.30);  *1-50% relative reduction equates to an absolute of 0.15-0.30 based on SoC mortality of 30%;
output;
end;
run;
data save_sim.Prior_2000g; set weighted_2000g; run;
```

Appendix Table S4B: Study trial simulations for infants ≤2000 g

```
**********************************************************************;
* Assurance calculations for 'Go' decision if neonatal mortality reduction >=15%      *;
* and assurance calculations for 'Consider' decision if reduction is 10-15%              *;
**********************************************************************;
data assurance_2000g;
 set weighted_200g;;

        Diff=placebo-prior;
           n = 526;              * n per arm *;

      p1 = placebo;
      p2 = prior;
      pbar = (p1+p2)/2;
      se = sqrt(2*(pbar*(1-pbar)/n));
      z_alpha = probit(0.5);

      z_den=SQRT((p1*(1-p1))+(p2*(1-p2)));

      z_num_15=(SQRT(n))*(p1-p2-0.045);  * 15% relative reduction (of 0.3) equates to 0.045*;

      z_15=(z_num_15/z_den)-z_alpha;

*******************************************************************************;
*The PROBNORM function returns the probability that an observation from the standard normal   *;
* distribution is less than or equal to x                                                          *;
*******************************************************************************;
      power_15_diff=probnorm(z_15);

      z_num_10=(SQRT(n))*(p1-p2-0.03);  * 10% relative reduction (of 0.3) equates to 0.03*;

      z_10=(z_num_10/z_den)-z_alpha;


*******************************************************************************;
*The PROBNORM function returns the probability that an observation from the standard normal *;
*distribution is less than or equal to x                                                          *;
*******************************************************************************;
        power_10_diff=probnorm(z_10);

        assurance_go=power_15_diff;

      assurance_consider=power_10_diff-power_15_diff;

run;

PROC means data=assurance_2000g n mean;
        TITLE1 'Assurance for babies <=2000g';
        TITLE2 'Assuming 48% probability that emollient will have an effect
above SoC';
        TITLE3 'Emollient effect believed to result in a relative reduction
of 1-50%';
        TITLE4 'Uniform distribution used to fit effect curve and Normal
distribution to fit no effect curve';
      VAR PRIOR p1 p2 assurance_go assurance_consider;
RUN;
```

Appendix Table S4C: Output for Study trail simulations for infants ≤2000 g

Assurance for babies <=2000g
Assuming 48% probability that emollient will have an effect above SoC
Emollient effect believed to result in a relative reduction of 1-50%
Uniform distribution used to fit effect curve and Normal distribution to fit no effect curve

The MEANS Procedure

Variable	N	Mean
prior	1000000	0.2639420
p1	1000000	0.3000000
p2	1000000	0.2639420
assurance_go	1000000	0.3660240
assurance_consider	1000000	0.0897515
